# Antifungal Th Immunity: Growing up in Family

**DOI:** 10.3389/fimmu.2014.00506

**Published:** 2014-10-15

**Authors:** Monica Borghi, Giorgia Renga, Matteo Puccetti, Vasileios Oikonomou, Melissa Palmieri, Claudia Galosi, Andrea Bartoli, Luigina Romani

**Affiliations:** ^1^Pathology Section, Department of Experimental Medicine, University of Perugia, Perugia, Italy; ^2^Polo GGB, Perugia, Italy

**Keywords:** Th cell subsets, immunity, tolerance, fungi

## Abstract

Fungal diseases represent an important paradigm in immunology since they can result from either the lack of recognition or over-activation of the inflammatory response. Current understanding of the pathophysiology underlying fungal infections and diseases highlights the multiple cell populations and cell-signaling pathways involved in these conditions. A systems biology approach that integrates investigations of immunity at the systems-level is required to generate novel insights into this complexity and to decipher the dynamics of the host–fungus interaction. It is becoming clear that a three-way interaction between the host, microbiota, and fungi dictates the types of host–fungus relationship. Tryptophan metabolism helps support this interaction, being exploited by the mammalian host and commensals to increase fitness in response to fungi via resistance and tolerance mechanisms of antifungal immunity. The cellular and molecular mechanisms that provide immune homeostasis with the fungal biota and its possible rupture in fungal infections and diseases will be discussed within the expanding role of antifungal Th cell responses.

## Fungal Infections and Diseases in the Metagenomics Era: A Reappraisal

Fungi can interact with their hosts (plants, animals, or human beings) in multiple ways, establishing symbiotic, commensal, or pathogenic relationships. Most fungi, such as *Aspergillus fumigatus* and *Cryptococcus neoformans*, and the thermally dimorphic fungi are ubiquitous in the environment, and human beings are exposed by inhaling spores or small yeast cells. In addition, more than 400 species of fungi associated with human beings have been identified (1). In this case, co-evolution of commensals, such as *Pneumocystis jirovecii, Malassezia* spp., and *Candida albicans*, with their mammalian hosts implicates the existence of sophisticated mechanisms to antagonize immunity in order to survive. Once considered pathogenic microbes, the commensal fungal microbiota is now an important component of the human intestinal ecosystem. Indeed, despite the intimate contact of fungi with the human host, fungal diseases in immunocompetent hosts are fairly uncommon, indicating that low-virulence fungi have evolved particular adaptation mechanisms that allow them to persist relatively unnoticed by the immune system (2). This “peaceful” coexistence may digress into overt disease under conditions of immune deregulation, such as in primary immunodeficiency human immunodeficiency virus infection and as a result of immunosuppressive therapies (2). In addition, invasive fungal diseases continue to be a serious problem in patients with hematologic disorders, solid, and hematopoietic organ transplantation as well as in non-high-risk, *sensu strictu*, patients, such as patients with *Mycobacterium tubercolosis* infection, hyper IgE syndrome, and anti-TNF-alpha therapy (3).

The increasing understanding of the importance of the microbiota in shaping the host immune and metabolic activity has rendered fungal interactions with the host and its microbiome more complex than previously appreciated (4) (**Box 1**). Indeed, the complex interactions between fungal and bacterial commensals, either directly or through the participation of the host immune system, all impact on the pathophysiology of a number of inflammatory disease that, in turn, may lead to secondary fungal infections (5, 6). Evidence is accumulating to support the exciting concept that the interaction between different biomes and between the host and the mycobiome are critical in the pathogenesis of fungal infections and other human diseases (1, 7, 8). Here, we will discuss recent findings on host- and microbial-dependent mechanisms of immune homeostasis with the fungal biota and its possible rupture in fungal infections and diseases.

Box 1 **The mycobiome at the host/microbiome interface**

. The development of culture-independent methods has expanded our knowledge of the mycobiomes found in different body sites, their interface with other biomes, and their association with human health and diseases (1). Alterations in the mycobiome are frequently reported to be associated with various diseases such as cystic fibrosis (9), inflammatory bowel diseases (6, 10, 11), atopic dermatitis (12), or mucocutaneous candidiasis (13). However, it remains to be elucidated whether this variation is primary or secondary to an imbalanced bacterial microbiome. Indeed, interactions of fungi with bacteria *in vitro* have been described [reviewed in Ref. (6)] as well as the clinical relevance of these interactions (14), such as the occurrence of intractable candidiasis in association with antibiotic-induced dysbiosis (15) and of mixed fungal–bacterial species in biofilms (14). Fungal–bacterial interactions can be antagonistic, synergistic, or symbiotic; regardless, they influence the physiological characteristics and survival of either one partner and, consequently, impact on host immune reactivity. Variations in the mycobiome can also be secondary to dysregulated host immune reactivity. The traditional view of a single direction by which bacteria stimulates the immune system, leading to inflammation or autoimmune disorders, has been challenged by a more complex view; the gut immune system does not simply protect from pathogens, but is actively involved in the maintenance of a rich and healthy community of gut bacteria (16). Faults in the immune regulation lead to changes in the bacterial community that in turn feed back into the immune system. Similar to the microbiome, the host/mycobiome interactions also lead to mutual influences. Not only is the host affecting the mycobiome composition and variations, by means of genotype, physiology, immune system, and lifestyle, but also the fungal microbiota may contribute to the balance of inflammation and tolerance at local mucosal surfaces and at distal sites (17).

## Resistance and Tolerance Mechanisms of Antifungal Immunity

As the immune system has evolved to accommodate colonization by symbiotic microbes while retaining the capacity to oppose their infectivity, a fine balance between pro- and anti-inflammatory signals is a prerequisite for a stable host/fungal relationship, the disruption of which may lead to pathological consequences. Indeed, despite the occurrence of severe fungal infections in immunocompromised patients, clinical evidence indicates that fungal diseases also occur in the setting of a heightened inflammatory response, in which immunity occurs at the expense of host damage and pathogen eradication (18). A number of fungal diseases are critical examples of such bidirectional influences between infection and immune-related pathology, a condition that highlights the bipolar nature of the inflammatory process in infection. Early inflammation prevents or limits infection, but an uncontrolled response may eventually oppose disease eradication. This conceptual principle is best exemplified by the occurrence of severe fungal infections in patients with chronic granulomatous disease (19), cystic fibrosis (20), or with immune reconstitution inflammatory syndrome (IRIS) (21), an entity characterized by local and systemic inflammatory reactions that can result in quiescent or latent infections manifesting as opportunistic mycoses. Chronic mucocutaneous candidiasis (CMC) and chronic disseminated candidiasis also belongs to the spectrum of fungus-related IRIS (22). Thus, an immune response that limits both fungal infectivity and host collateral damage is required to maintain a homeostatic environment (23). This dual role has recently been accommodated within the conceptual framework of a two-component antifungal immune response, i.e., resistance – the ability to limit fungal burden – and tolerance – the ability to limit the host damage caused by either the immune response or other mechanisms (2). Resistance is meant to reduce pathogen burden through innate and adaptive immune mechanisms, whereas a plethora of tolerance mechanisms, despite less known relative to resistance mechanisms, protect the host from immune- or pathogen-induced damage (24).

## Mechanisms of Antifungal Resistance

Innate immune mechanisms are used by the host to respond to a range of fungal pathogens in an acute and conserved fashion. The constitutive mechanisms of innate defense are present at sites of continuous interaction with fungi and include the barrier function of body surfaces and the mucosal epithelial surfaces of the respiratory, gastrointestinal, and genitourinary tracts. Microbial antagonism, defensins, collectins, and the complement system realize the strict fungus specificity of the constitutive mechanisms and provide opsonic recognition. Multiple cell populations and cell-signaling pathways are involved in the antigen-independent recognition of fungi by PRRs (2, 25). Both murine and human studies have confirmed the association of susceptibility to fungal infections and diseases with genetic deficiency of selected PRRs (2). Because PRRs not only mediate downstream intracellular events related to fungal clearance but also participate in activation of adaptive immunity, deficiencies on innate immune genes also reverberate on the type and quality of the adaptive immune response, including effector CD4+ T helper (Th), regulatory T (Treg), and CD8+ T-cells (2, 25–27).

### Dendritic cells

It is well established that the adaptive immune response, in particular that of T-cells, plays a pivotal role in antifungal host defense (2, 25). Dendritic cells (DCs) play a key role in promoting T-cell differentiation and responses to ubiquitous or commensal fungi. Studies have shown that lung DCs can transport fungal antigens to the draining lymph nodes (28, 29), where they orchestrate T-cell activation and differentiation into effector cells. Through elaboration of distinct sets of cytokines and other mediators, DCs have the unique ability to elicit a robust T-cell response that can be either tolerogenic or pro-inflammatory in nature, based on anatomical location and local metabolic environment. The whole-genome transcriptional analysis of DCs stimulated with fungi evidenced the presence of peculiar transcriptional programs governing the recognition of fungi (30).

These include common signaling pathways involving Syk kinase, Card9 and NF-κB downstream CLRs and ERK kinase, PI3K/Akt downstream TLRs for Th1/Th2/Th17 priming by conventional, inflammatory DCs, as well as p38/TRIF/STAT3 for Treg priming by plasmacytoid DCs (2, 31). In a mutual interaction, the host and the fungus control each other to avoid potential harmful inflammatory response. The ability of a given DC subset to respond with flexible activating programs and activation of distinct intracellular signaling pathways to the different PRR/fungal molecules’ combinations confers unexpected plasticity to the DC system and pivotally contributes in shaping adaptive Th cells responses in infection and vaccination. The capacity of DCs to initiate different adaptive antifungal immune responses also depends upon specialization and cooperation between DC subsets (32). The multiple, functionally distinct, receptor/signaling pathways in DCs, ultimately affecting the local Th/Treg balance, are likely successfully exploited by fungi from commensalism to infection (33).

### Th1 cells

CD4+ Th cells exist in a variety of epigenetic states that determine their function, phenotype, and capacity for persistence, and form long-term immune memory (34). Well-balanced Th1 and Th17 cell responses are crucial in antifungal immunity and facilitate phagocytic clearance of fungal recognition, mainly through release of cytokines such as TNF-α, IFN-γ, and IL-17A and IL-17F (Table 1). These cytokines stimulate the disparate antifungal effector functions of phagocytes, as well as the generation of optimal T-cell-dependent immunity (2, 25). A dominant Th1 response correlates with the expression of protective immunity to fungi (2, 35) and vaccines (36, 37). Through the production of the signature cytokine IFN-γ and help for opsonizing antibodies, the activation of Th1 cells is instrumental in the optimal activation of phagocytes at sites of infection. Therefore, the failure to deliver activating signals to effector phagocytes may predispose patients to overwhelming infections, limit the therapeutic efficacy of antifungals and antibodies, and favor fungal persistency (2). Patients who are deficient in IL-12Rβ are susceptible to CMC, which is frequently recurrent or persistent (38), as well as to deep paracoccidioidomycosis (39).

**Table 1:** **CD4^+^ Th cell subsets in fungal infections**.

Th cells		Cytokines	Functions
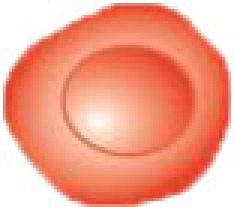	Th1	IFN-γ/TNF-α	Fungal clearance
			Inflammation
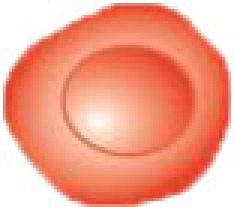	Th17	IL-17A/IL-17F	Defensins, neutrophil recruitment
			Inflammation
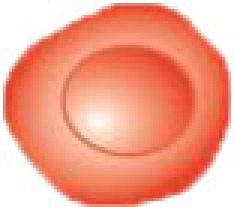	Th22	IL-22	Defensins
			Tissue homeostasis
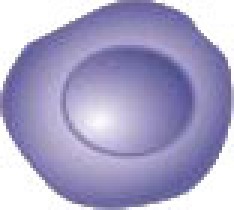	Th2	IL-4/IL-13	Humoral response
			Allergy
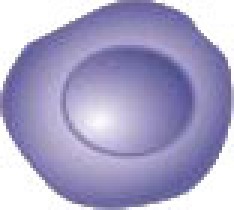	Th9	IL-9/IL-10	Tissue inflammation
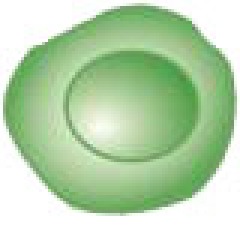	Treg	IL-10/TGF-β	Low inflammation
			Immunosuppression
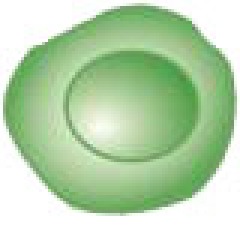	Tr1	IL-10	Low immunopathology

### Th17 cells

Th17 are present in the human T-cell memory repertoire to fungi (2) and inborn errors of human IL-17 immunity underlie susceptibility to CMC (40) in which both Th17 (41) and Th1 (38, 42, 43) responses are defective. Combined deficiency of the Th1 and Th17 pathway predisposes to fungal diseases (44, 45), thus emphasizing the important role played by both pathways in resistance against fungi. This could be explained with the notion that Th17 cells, although found early during the initiation of an immune response, are involved in a broad range of Th1-, Th2- and Treg-dominated immune responses (2, 46). In terms of effector functions, the ability of IL-17A to mobilize neutrophils and induce defensins may contribute to a prompt and efficient control of the infection at the different body sites. In respiratory fungal infections, Th17 cell are dispensable for resistance to the primary infection caused by *A. fumigatus* (47), but are required for vaccine-induced immunity against systemic mycoses endemic to North America (48). Thus, both Th17 and Th1 (27) cells are required for vaccine immunity to respiratory fungal pathogens.

It is intriguing that Th17 responses are down regulated by *C. albicans* (49). Regardless of the contribution of this phenomenon to infection or commensalism, this finding suggests that Th17 responses are finely tuned by fungi, as the failure to downregulate Th17 may eventually result in chronic inflammation and failure to resolve the infection (47, 50). The mechanisms that linked inflammation to chronic infection have been credited to the offending potential of IL-17A that, although promoting neutrophil recruitment, impeded the timely restriction of neutrophil inflammatory potential (51) while directly promoting fungal virulence (52). Thus, the Th17 pathway could be involved in the immunopathogenesis of chronic fungal diseases where persistent fungal antigens may maintain immunological dysreactivity. This may happen in autoimmune polyendocrine syndrome type 1 patients (APS-1) and Aire-deficient mice (53) where an excessive Th17 reactivity was observed. This finding apparently conflicts with the presence of autoantibodies against IL-22, IL-17A, and IL-17F observed in these patients (54, 55). Although correlated to susceptibility to CMC, these antibodies were also present in patients without CMC. In addition, despite the presence of antibodies to type I IFN, APS-I patients do not appear prone to recurrent viral infections. It has instead been shown that autoantibodies to pro-inflammatory cytokines may act as beneficial autoimmunity in their ability to dampen pro-inflammatory mediators and restrict self-destructive immunity (56).

### Th2 cells

IL-4 and IL-13 act as the most potent proximal signal for commitment to Th2 reactivity that, by dampening protective Th1 responses and promoting the alternative pathway of macrophage activation, favors fungal persistence, allergy, and disease relapse. Limiting IL-4 production restores antifungal resistance (2) (Table 1). In atopic subjects and neonates, the suppressed DTH response to fungi is associated with elevated levels of antifungal IgE, IgA, and IgG. In CF patients, heightened Th2 reactivity associates with allergic bronchopulmonary aspergillosis and is sensitive to vitamin 3 (57). However, alternatively activated macrophages may have a protective role in defense against some respiratory fungi (58, 59) and Th2-dependent humoral immunity may afford some protection, in part by promoting Th1 immunity (60) and by altering fungal gene expression and intracellular trafficking (61–63). The efficacy of certain vaccines that elicit protective antibody strongly indicates that antibody responses can make a decisive contribution to host defense to fungi (61).

### Th9 cells

The realization that Th effectors can produce various other cytokines alone or in combination in patterns not fitting the preconceived definitions of Th1/Th2 or Th17 subsets has led to the description of additional Th cell lineages, including Th9 and Th22. Initially thought to be a Th2-specific cytokine by virtue of its role in the pathogenesis of asthma, IgE class switch recombination, and resolution of parasitic infections, IL-9 is now considered to be the product of a distinct Th subset, the Th9 (64). Despite its relationship with other subsets, such as Th2, Th17, and Treg cells, Th9 cell subset can mediate tumor immunity and participate in autoimmune and allergic inflammation. Recently, human memory Th9 cells were found to be skin tropic or skin resident. Human Th9 cells co-expressed TNF-α and granzyme B, lacked coproduction of Th1/Th2/Th17 cytokines, and many were specific for *C. albicans*. IL-9 production preceded the upregulation of other inflammatory cytokines, such as IFN-γ, IL-13, and IL-17. IL-9-producing T-cells were increased in the skin lesions of psoriasis, suggesting that these cells may contribute to human inflammatory skin disease in the presence of *Candida* (65). Recent findings demonstrated that IL-9 is predominantly produced *in vivo* by a novel subset of innate lymphoid cells termed ILC2 (66). It has been proposed that IL-9 might have a regulatory and prosurvival function for many lymphoid and myeloid cells (67). Our recent evidence suggests that different types of ILCs are defective in IL-9-deficient mice infected with either *C. albicans* or *A. fumigatus*, and this profoundly affects the outcome of either infection and the associated pathology (unpublished observations) (Table 1).

### Th22 cells

Th22 cells producing only IL-22 but neither IFN-γ nor IL-17A have been identified in human beings (68). They are induced in the presence of TNF-α and IL-6 and require ligation of aryl hydrocarbon receptor (AhR). Th22 cells via IL-22 influence the function of mesenchymal and epithelial cells and have been implicated in the dermatopathology of psoriasis and atopic dermatitis (69, 70). Memory *C*. *albicans*-specific IL-22+ CD4+ cells are present in human beings and defective in patients with CMC (71). Recent evidence indicates that IL-22 may play a crucial role in the innate immune resistance and local protection in mucocutaneous fungal diseases (72–74). Through the exploitation of primitive anti-fungal defense mechanisms, IL-22 was crucially involved in the control of *Candida* growth at mucosal sites in conditions of Th1 and Th17 deficiency (72, 74). Produced by ILC3 cells expressing AhR, IL-22 directly targeted gut epithelial cells to induce STAT3 phosphorylation and the release of S100A8 and S100A9 peptides known to have anti-candidal activity and anti-inflammatory effects (72, 74). Thus, due to dominant-negative mutations of STAT3, patients with autosomal dominant hyper-IgE syndrome have a defective Th17 (41) that is likely amplified on ECs where STAT3 mutation compromises the IL-22 effects. IL-22 also mediates antifungal resistance and epithelial protection in experimental and human vulvovaginal candidiasis (VVC) as well as in recurrent VVC (RVVC). In RVVC, functional genetic variants in IL22 genes were found to be associated with heightened resistance to RVVC, and they correlated with increased local expression of IL-22 (74). Thus, IL-22+ cells, employing ancient effector mechanisms of immunity, may represent a primitive mechanism of resistance against fungi under a condition of limited inflammation (Table 1). The fact that IL-22 production in the gut is driven by commensals (see below) also provides novel mechanistic insights on how antibiotic-related dysbiosis may predispose to candidiasis (75).

## Mechanisms of Tolerance

### Treg cells

The exposure to fungi requires the generation of a controlled immune response in the host that recognizes and controls them, limits collateral damage to self-tissues, and restores a homeostatic environment. A number of clinical observations suggest an inverse relationship between IFN-γ and IL-10 production in patients with fungal infections. High levels of IL-10, negatively affecting IFN-γ production, are detected in chronic candidal diseases, in the severe form of endemic mycoses, and in neutropenic patients with aspergillosis. Thus, high levels of IL-10 have been linked to susceptibility to fungal infections (76). However, given its prominent effect on resolution of inflammation, IL-10 production may be a consequence, rather than the cause, of the infection. This predicts that, in the case of chronic fungal infections dominated by non-resolving, persisting inflammation, IL-10 produced by Treg cells acts as homeostatic host-driven response to keep inflammation under control. Treg cells with anti-inflammatory activity have been described in fungal infections of both mice and human beings (2, 25). In experimental fungal infections, inflammatory immunity and immune tolerance in the respiratory or the gastrointestinal mucosa were all controlled by the coordinate activation of different Treg cell subsets, exerting a fine control over effector components of innate and adaptive immunity. Seen in this context, the Treg/IL-10 axis is a dangerous necessity, the failure of which may lead to detrimental inflammation. However, as the Treg responses may handicap the efficacy of protective immunity, the consequence of Treg activity is less damage to the host but also fungal persistence and immunosuppression, eventually (Table 1). Thus, by controlling the quality and magnitude and effector innate and adaptive responses, the spectrum of Treg cell activities may go from “protective tolerance,” defined as a host’s response that ensures survival of the host in a trade-off between sterilizing immunity and its negative regulation limiting pathogen elimination to overt immunosuppression. Taking a step further, this suggests that the interaction between fungi and the host immune status may determine their position from commensals to pathogens, and this position can change continuously. The salutary effects of Treg cells may go beyond their anti-inflammatory properties, to include the polarization of protective Th17 cells (46).

### Tr1 cells

T regulatory Type 1 (Tr1) cells are adaptive Treg cells characterized by the ability to secrete high levels of IL-10. Since their discovery, Tr1 cells have been proven to be important in maintaining immunological homeostasis and preventing T-cell-mediated diseases. Tr1 cells suppress T- and DC-dependent responses primarily via the secretion of IL-10 and TGF-β, release of granzyme B and perforin, and by disrupting the metabolic state of T effector cells. Tr1 cells have been demonstrated to have a role in infectious diseases, autoimmunity, and transplant rejection in different pre-clinical disease models and in patients (77). It has recently been shown that Tr1 cells play a distinct, yet complementary role, in response to *A. fumigatus* in human beings and mice. Tr1 cells specific for an epitope derived from the cell wall glucanase Crf-1 of *A. fumigatus* (Crf-1/p41) were identified in healthy human beings and mice after vaccination with Crf-1/p41+ zymosan. These cells produced high amounts of interleukin IL-10 and suppressed the expansion of antigen-specific T-cells *in vitro* and *in vivo*, thus limiting immunopathology (Table 1). *In vivo* differentiation of Tr1 cells was dependent on the presence of AhR, c-Maf, and IL-27. In comparison to Tr1 cells, Foxp3+ induced Treg that recognize the same epitope were induced in an interferon gamma-type inflammatory environment and more potently suppressed innate immune cell activities. These data provide evidence that Tr1 cells are involved in the maintenance of antifungal immune homeostasis, and most likely play a distinct, yet complementary, role compared with Foxp3+ Treg cells (78).

## Tryptophan Metabolism

The enzyme indoleamine 2,3-dioxygenase 1 (IDO1) and its downstream catabolites sustain the delicate balance between Th1/Th17 pathways and Treg cells, by providing the host with adequate protective immune mechanisms without necessarily eliminating the pathogen or causing undesirable tissue damage (79). As a result of their ability to induce differentiation of Treg cells and inhibit Th17 cells, IDO1 is critical to cell lineage commitment in experimental fungal infections and contributes to the overall outcome of inflammation, allergy, and Th17-driven inflammation in these infections. Under these circumstances, the Th17 pathway, by inhibiting tryptophan catabolism, may instead favor pathology and provides evidence accommodating the apparently paradoxical association of chronic inflammation with fungal disease (19). IDO1 is a “metabolic” enzyme conserved through the past 600 million years of evolution. Initially recognized in infection because of antimicrobial activity (“tryptophan starvation” of intracellular parasites), IDO1 is now widely recognized as suppressor of acute inflammatory responses and regulator of mammalian immune homeostasis (79). Not surprising, IDO1 may represent an evasion mechanism for microbes that establish commensalism or chronic infection (79). In their capacity to induce Tregs and inhibit Th17, IDO1-expressing DCs and epithelial cells and kynurenines revealed an unexpected potential in the control of inflammation, allergy, and Th17-driven inflammation in these infections (51, 80).

## Microbiota Regulation of Resistance and Tolerance to Fungi

Commensal-driven mucosal responses are upregulated in IDO1 deficiency (81) and IL-22 responses are upregulated in conditions of defective adaptive immunity (72) and IDO deficiency (75). AhR is a ligand-activated transcription factor that mediates IL-22 production (82). A variety of indole derivatives act as endogenous ligands for AhR (83) and are generated through conversion from dietary tryptophan by commensal intestinal microbes (84). Recent evidence has shown that AhR is involved in the (patho)physiology of skin including the regulation of skin pigmentation, photocarcinogenesis, and skin inflammation (85, 86). Of interest, the ability of *Malassezia*-derived indoles to activate AhR correlated with local immunoregulation (87) and pathogenicity in seborrheic dermatitis (88). Similarly, metabolomics has revealed that bioactive indoles with Ahr agonist activity are also present in mice with candidiasis (75). Thus, the trpyptophan metabolism pathway is exploited by commensals and the mammalian host to increase fitness in response to fungi via induction of resistance and tolerance at the skin and mucosal surface. The new findings support a model in which the IL-22 axis controls the initial fungal growth (i.e., resistance) and epithelial cells homeostasis likely exploiting primitive anti-fungal effector defense mechanisms. In contrast, the exploitation of the IFN-γ/IDO 1 axis for functional specialization of antifungal regulatory mechanisms (i.e., protective tolerance) may have allowed the fungal microbiota to co-evolutes with the mammalian immune system, survives in conditions of high-threat inflammation, and prevents dysregulated immunity (79). The two pathways, although non-redundant, are reciprocally regulated and compensate each other in the relative absence of either one (72), consistent with the theme that adaptive immunity depends on innate immunity but innate immunity requires adaptive regulation. This finding not only helps to explain the association of fungal infections with dysbiosis but also points to the essential help the microbiota may provide in fungal colonization and pathogenicity in immunodeficient patients.

## Conclusion

Vertebrates have co-evolved with microorganisms resulting in a symbiotic relationship, which plays an important role in shaping host immunity. However, intestinal inflammation also dictates the composition of gut-associated microbial communities (89), a finding indicating the reciprocal influence of the microbiota and the mammalian immune status. The mycobiome is not an exception to the rule. The activation of different Th cells with distinct effector and immunoregulatory functions may impact differently on the local mycobiome composition. Indeed, the findings that fungi oppositely react to IFN-γ (90) or IL-17A (52), in terms of growth and virulence, suggest that the local Th environment may contribute to the diversity of the mycobiome at different body sites. Ultimately, fungi have evolved a contingency-based system during co-evolution to adapt to host immunity and persist in an inflammatory host environment. In turn, this feeds back into the host immune fitness. For instance, manipulation of the regulatory network of the host by the fungal microbiota, resulting in the activation of Treg-dependent immune tolerance, is a mechanism to ensure fungal survival and commensalism at different body sites, as well as local immune tolerance (76, 91, 92). Thus, challenging existing paradigms with new perspectives from the crosstalk between fungi, the immune system, and the microbiota will eventually lead toward the development of multi-pronged therapeutic approaches for mucosal and systemic fungal diseases.

## Conflict of Interest Statement

The authors declare that the research was conducted in the absence of any commercial or financial relationships that could be construed as a potential conflict of interest.

## Abbreviations

AhR, aryl hydrocarbon receptor; APS-1, autoimmune polyendocrine syndrome type 1 patients; CF, cystic fibrosis; CLR, C-type lectin receptors; CMC, chronic mucocutaneous candidiasis; DCs, dendritic cells; DTH, delayed type hypersensitivity; IDO1, indoleamine 2,3-dioxygenase 1; ILC3, innate lymphoid cells 3; IRIS, immune reconstitution inflammatory syndrome; PRRs, pattern recognition receptors; RVVC, recurrent VVC; Th, T helper; Treg, regulatory T-cells; VVC, human vulvovaginal candidiasis.
